# A Hybrid Nanogenerator Based on Rotational-Swinging Mechanism for Energy Harvesting and Environmental Monitoring in Intelligent Agriculture

**DOI:** 10.3390/s25165041

**Published:** 2025-08-14

**Authors:** Hao Qian, Yuxuan Zhou, Zhi Cao, Tian Tang, Jizhong Deng, Xiaoqing Huo, Hanlin Zhou, Linlin Wang, Zhiyi Wu

**Affiliations:** 1 College of Engineering, Zhejiang Normal University, Jinhua 321004, China; qianhao@zjnu.edu.cn; 2 College of Civil Engineering and Architecture, Zhejiang University, Hangzhou 310058, China; zhouyuxuan@binn.cas.cn; 3 Beijing Institute of Nanoenergy and Nanosystems, Chinese Academy of Sciences, Beijing 101400, China; caozhi@binn.cas.cn (Z.C.); tangtian@binn.cas.cn (T.T.); dengjizhong@binn.cas.cn (J.D.); huoxiaoqing@binn.cas.cn (X.H.); zhouhanlin@binn.cas.cn (H.Z.); 4 International Institute for Interdisciplinary and Frontiers, Beihang University, Beijing 100191, China; wuzhiyi@buaa.edu.cn

**Keywords:** triboelectric nanogenerator, electromagnetic generator, intelligent agriculture, environmental monitoring, self-powered systems

## Abstract

With the rapid growth of the Internet of Things, intelligent agriculture is becoming increasingly important. Traditional agricultural monitoring methods, which rely on fossil fuels and complex wiring, hinder progress. This work introduces a hybrid nanogenerator based on a rotational-swinging mechanism (RSM-HNG) that combines triboelectric nanogenerators (TENGs) and electromagnetic generators (EMGs) for efficient wind energy harvesting and smart agriculture monitoring. The parallelogram mechanism and motion conversion structure enable the stacking and simultaneous contact-separation of multiple TENG layers. Moreover, it allows the TENG and EMG units to operate simultaneously, which improves energy harvesting efficiency and extends the system’s lifespan compared to traditional disc-based friction wind energy harvesting methods. With four stacked layers, the short-circuit current of the TENG increases from 16 μA to 40 μA, while the transferred charge rises from 0.3 μC to 1.5 μC. By optimizing the crank angle, material selection, and substrate structure, the output performance of the RSM-HNG has been significantly enhanced. This technology powers a self-sustaining wireless monitoring system for temperature, humidity, an electronic clock, and road guidance. The RSM-HNG provides continuous energy for smart agriculture, animal husbandry, and environmental monitoring, all driven by wind energy. It holds great potential for regions with abundant wind resources but limited electricity access, offering valuable applications in these areas.

## 1. Introduction

With growing global energy demand, fossil fuels (e.g., coal, oil, and natural gas) continue to be the main source of energy consumption in most countries [1,2,3]. However, the widespread use of fossil fuels not only results in significant carbon dioxide emissions, exacerbating global warming, but also produces harmful gases such as sulfur oxides and nitrogen oxides. These pollutants not only degrade air quality but also contribute to the formation of acid rain, which can cause severe damage to ecosystems [4,5,6]. In contrast, wind energy, as a clean and sustainable energy source, offers advantages such as wide geographical adaptability, low operating costs, and virtually zero emissions, which can effectively mitigate the negative environmental impact [7,8,9]. In recent years, with the continuous development of wind power generation technology, the efficiency and economic viability of wind energy utilization have been significantly improved. Especially in wind resource-rich agricultural areas, wind energy has become one of the main sources of energy supporting agricultural production and intelligent monitoring systems [10,11,12].

The triboelectric nanogenerator (TENG), as an emerging energy harvesting technology, holds great potential for applications in wind energy [13,14,15,16,17]. The TENG utilizes the triboelectric effect and charge transfer principle [18] to convert mechanical energy, such as wind energy, into electrical energy. The advantages of this technology include its simplicity, low cost, high efficiency in capturing weak ambient energy, and independence from traditional power grids or fuel supplies [19,20]. By combining the electromagnetic generator (EMG) and the TENG, the high-current, low-voltage characteristics of the EMG and the low-voltage, high-current characteristics of the TENG can be effectively integrated to further enhance energy harvesting efficiency [21,22,23]. Several studies have reported generators for wind energy harvesting, typically operating in vibration mode [24,25,26], rotary mode [27,28,29], sliding mode [30], etc. Wang et al. proposed a Hybrid Triboelectric–Electromagnetic–Electric Field Energy Harvester (TEE-HEH), in which friction between a rotating rotor and stator generates electrical energy for environmental monitoring systems in smart grids [31]. Another example is Zhao et al., who designed a hybridized triboelectric-electromagnetic nanogenerator capable of providing stable power to wireless sensor nodes and Bluetooth modules in real time via wind energy [32]. However, these wind-driven generators are susceptible to significant wear and tear and exhibit unstable output.

Agricultural automation is an inevitable trend driven by the demands of the modern era, and the sensors used in this field require a reliable power supply. Traditional power methods typically rely on wires or batteries, which are not only complex to install but also require periodic replacement. Most wind energy-harvesting devices employ rotating TENG structures and rotating EMG structures in a layered configuration. This work presents a hybrid nanogenerator based on a rotational-swinging mechanism (RSM-HNG), which captures wind energy using a wind cup and converts the wind-driven rotational motion into swinging motion via a rotary-swing motion conversion mechanism. The rotational motion drives the electromagnetic generator (EMG) to generate electrical energy, while the oscillating motion drives the triboelectric nanogenerator (TENG) to produce electrical energy. The parallelogram structure of the TENG unit allows for multiple stackings to increase output power. This design not only extends the lifespan of the device but also ensures its output performance. The RSM-HNG utilizes a contact-separation mode of power generation to enhance service life [33], optimizing the crank angle, material, and substrate structure by selecting a 7.5° crank, an FEP-Nylon material combination, and a substrate soft-contact treatment to improve output performance. At a speed of 90 rpm, the TENG unit achieves a peak power of 11.7 mW, while the EMG unit achieves a peak power of 30.24 mW. An intelligent agriculture monitoring system based on RSM-HNG was established to verify its potential for wind-driven applications, capable of realizing Bluetooth environmental monitoring, an electronic clock display, and nighttime road indication.

## 2. Experimental

### 2.1. Fabrication of the RSM-HNG

The fabrication was completed using a laser cutting machine (CMA6040-DZ, Shenzhen, China) and a precision engraving machine (CNC4030, Dongguan, China). The main components include the base plate, Z-bar, and long connecting rod. These parts are made of PMMA. The base plate measures 140 mm by 190 mm, and a 1 mm thick sponge of 140 mm by 190 mm is adhered to the base plate. A 50 µm thick copper foil of the same dimensions (140 mm by 190 mm) is pasted onto the sponge to serve as the electrode. A 10 µm thick Nylon layer of 140 mm by 90 mm is adhered to the electrode as the positive triboelectric material layer, and a 50 µm thick FEP layer of 140 mm by 90 mm is pasted onto the opposite end of the electrode as the negative triboelectric material layer. The Z-rod and the long connecting rod are connected using clamp nuts, and the Z-rod is fixed to the base plate with bolts and nuts.

The rotary-swinging mechanism is fabricated using a lathe (BB25, Shenzhen, China) and a 3D printer (BambuLab X1C, Shenzhen, China). Its main components include the oscillating shaft, triangular plate, bearing, rotary shaft, crank, and bearing bracket. The oscillating shaft and rotary shaft are made of aluminum alloy and are machined using a lathe, while the triangular plate, crank, and bearing bracket are fabricated through 3D printing.

The EMG unit is fabricated using a precision engraving machine (CNC4030, Shenzhen, China). Its main components include the magnet, electromagnetic coil, and magnet holder. The magnet is made of NdFeB material with dimensions of 48 mm by 24 mm by 5 mm. The electromagnetic coil is a square ring with a wire diameter of 0.06 mm and 2450 turns. Its external dimensions are 48 mm by 24 mm by 5 mm, with an inner hole size of 32 mm by 8 mm by 5 mm. The magnet holder is fabricated using the precision engraving machine, and the magnet is bonded into the hole of the magnet holder using hotmelt.

### 2.2. Electrical Measurement of the RSM-HNG

The drive for the RSM-HNG was provided by a rotary motor (PR02, Zurich, Switzerland), with wind generated by a commercial blower (MNT070088, Jinhua, China). Wind speed was measured using an anemometer (San-Liang RA620, Shanghai, China). The wireless sensor employed was a Xiaomi Bluetooth thermo-hygrometer (LYWSD03MMC, Beijing, China). Electrical performance was assessed using an electrometer (Keithley 6514, Cleveland, OH, USA), a signal acquisition system (NI USB-6346, Austin, TX, USA), and host computer software (LabVIEW 2018). A multimeter (Agilent U1242B, Beijing, China) was used to measure the capacitor voltage during the on-site wind energy collection process. A commercial push–pull sensor (SimBaTouch SBT674-1KG, Guangzhou, China) was used to measure the force required to drive the TENG unit.

## 3. Results and Discussion

### 3.1. Design and Working Principle of the RSM-HNG

**Figure 1:**
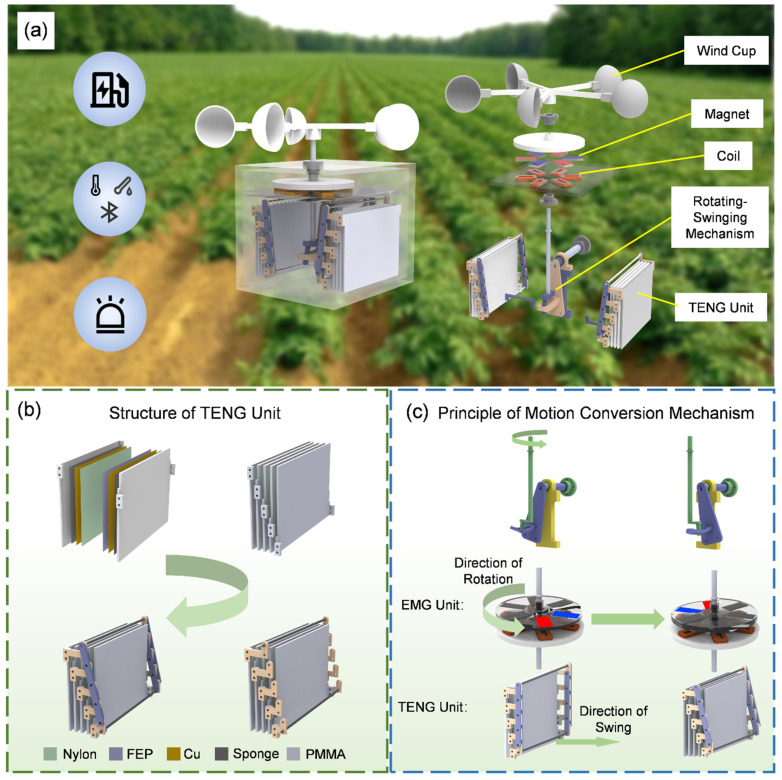
Application, structure, and mechanism of the RSM-HNG: (**a**) schematic illustration of the RSM-HNG application; (**b**) schematic representation of the TENG unit structure in the RSM-HNG; (**c**) working principle diagram of the rotary-swinging motion conversion mechanism.

Figure 1a illustrates the application scenarios of the RSM-HNG, which can be widely used in agriculture, particularly in outdoor agricultural settings. It can be driven by wind energy for efficient energy harvesting, enabling the development of a self-powered Bluetooth temperature and humidity sensor system, as well as a nighttime road indication system. Figure 1a also demonstrates an exploded view of the overall structure of the RSM-HNG, where the system consists of a wind cup, an EMG unit (consisting of coils and magnets), a rotary-swing motion conversion mechanism, and a TENG unit. Figure S1 in the Supplementary Material shows the physical photograph of the RSM-HNG. Figure S2 in the Supplementary Material shows the main dimensions of the RSM-HNG.

Figure 1b illustrates the structure of the TENG unit. The substrate is made of Polymethyl Methacrylate (PMMA), with a sponge attached to its surface as a cushioning layer, ensuring more adequate contact between the TENG unit and the substrate. The sponge layer is covered with a copper film as the electrode layer, which is then coated with a triboelectric material layer. Nylon is used as the positive friction material, while FEP serves as the negative friction material. By contacting and separating these two materials with different electronegativities, the TENG unit generates electrical output. The TENG unit consists of five substrates forming four groups of stacked TENGs, which are connected by a parallelogram mechanism made up of Z-bars and connecting rods to form a complete TENG unit. Thanks to the design of the parallelogram mechanism, the four groups of TENGs can synchronize contact and separation, optimizing the efficiency of energy harvesting and conversion.

Figure 1c illustrates the operational principle of the motion conversion mechanism and how it drives the EMG unit to work in conjunction with the TENG unit. Figure S3 in the Supplementary Material shows the structure of the EMG unit and the arrangement of the magnet. Firstly, the wind cup rotates the shaft under the influence of wind force. A crank is attached to the rotating shaft, and its rotation drives the spatial slide mechanism, generating a swinging motion. This transforms the input rotational motion into swinging motion. The operation of the EMG unit depends on the rotation of the shaft, and as the shaft rotates, the bracket with the magnets rotates accordingly, causing the coils to cut through the magnetic flux, thus generating electrical output. The drive of the TENG unit relies on a motion conversion mechanism. When the shaft rotates, the crank mounted at the bottom of the shaft rotates synchronously. The cylindrical rotor at the bottom of the crank fits into the slots of the triangular plate on the spatial slide, which is designed to constrain the movement of the rotor. When the crank rotates, the triangular plate generates a swinging motion, which drives the TENG unit to make contact and separate, ultimately resulting in electrical output. Through this design, the RSM-HNG efficiently utilizes wind energy for energy harvesting and powers various low-power devices, widely used in environmental monitoring and road guidance in agriculture.

**Figure 2:**
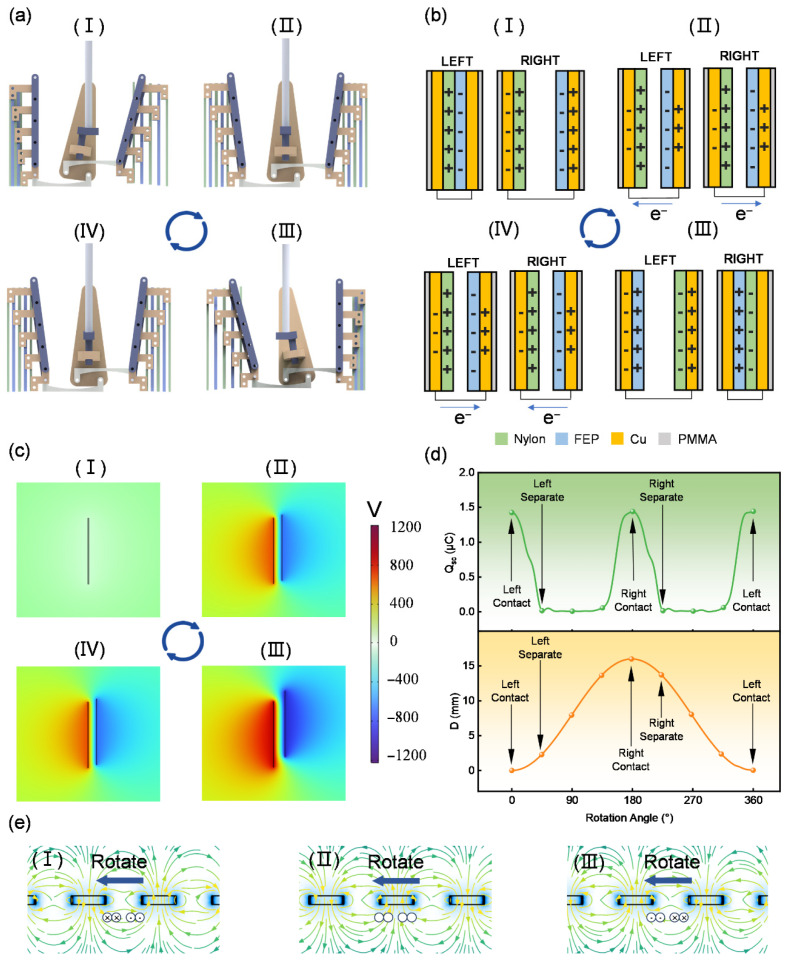
Schematic diagram of the operating principle of the TENG unit in the RSM-HNG: (**a**) cyclic operation process of the TENG unit. Step (I): left TENG contacts and right TENG separates; Step (II): left TENG separates and right TENG separates; Step (III): left TENG separates and right TENG contacts; Step (IV): left TENG separates and right TENG separates. (**b**) charge distribution during the cyclic operation of the TENG unit. Step (I): left TENG contacts and right TENG separates; Step (II): left TENG separates and right TENG separates; Step (III): left TENG separates and right TENG contacts; Step (IV): left TENG separates and right TENG separates. (**c**) simulation of potential distribution during the working cycle of the TENG unit. Step (I): left TENG contacts and right TENG separates; Step (II): left TENG separates and right TENG separates; Step (III): left TENG separates and right TENG contacts; Step (IV): left TENG separates and right TENG separates. (**d**) effect of the rotary axis angle on separation distance and QSC in the TENG unit; (**e**) simulation of magnetic field distribution and induced current during the working cycle of the EMG unit. Step (I): the coil is located in the middle of adjacent magnets; Step (II): Align the coil with the magnet; Step (III): Place the coil in the middle of adjacent magnets again.

Figure 2 illustrates the operating principle of the TENG unit in the RSM-HNG. As shown in Figure 2a, the TENG unit undergoes a complete cyclic process driven by the motion conversion mechanism. The rotating shaft of the rotating-swinging motion mechanism drives the crank to rotate, thus driving the slide groove swinging motion to perform a reciprocating swing around the shaft. Stage (I) in Figure 2a is defined as the 0° position of the rotational axis, corresponding to state (I) in Figure 2b, when the triboelectric material layer on the left side is in contact, generating a corresponding induced charge via the copper electrodes attached to the Fluorinated Ethylene Propylene (FEP) film. As the rotary axis rotates 90° from 0°, it drives the slotted oscillating member to swing to the right, which in turn drives the long connecting rod of the triboelectric nanogenerator to rotate. Under the constraint of the parallelogram mechanism, the triboelectric material layer achieves parallel separation and enters stage (II), corresponding to state (II) in Figure 2b. In this stage, the left triboelectric material layer begins to separate, and the two triboelectric materials beneath the copper electrode cause the electrons to move in the external circuit due to the difference in charge polarity distribution, resulting in the generation of current. The crank continues to rotate 90° to stage (III), corresponding to state (III) in Figure 2b, at which point the triboelectric material layer of the right triboelectric nanogenerator portion comes into contact, generating an induced charge on the copper electrode once again. Then, the crank rotates 90° to stage (IV), corresponding to state (IV) in Figure 2b, and the triboelectric material layer on the right side begins to separate, generating current in the external circuit once again. Finally, the crank rotates 90° back to stage (I), completing the full cyclic process. The rotation of the crank drives the sliding slot’s swinging motion, which in turn enables the cyclic contact–separation of the triboelectric material layer, causing the charge on the copper electrode to move continuously in the external circuit, thus generating a stable electrical output.

Figure 2c shows the COMSOL (Version 6.1) simulation of the TENG unit. Initially, in stage (I), the two triboelectric layers are in contact, and no potential difference is generated. In stage (II), the triboelectric layers begin to separate, and a potential difference is generated across the ends of the layers. As the separation distance increases and the system reaches stage (III), the potential difference between the two ends of the triboelectric layers reaches its maximum. Subsequently, as the separation distance decreases, the potential difference begins to decrease (stage IV), eventually returning to state (I) when the layers completely close, and the potential difference returns to zero, completing one cycle.

Figure 2d demonstrates the relationship between the rotation angle of the rotational axis and both the contact–separation distance (D) and the transferred charges (QSC) of the TENG unit. State (I) in Figure 2a is defined as 0°, at which point the left TENG unit is closed, and the transferred charges reach their maximum value. As the rotational axis turns, the separation between the triboelectric material layers of the TENG units begins, causing the transferred charge of the left-side TENG unit to gradually decrease to 0. When the rotational axis continues to rotate by 180°, it reaches state (III) in Figure 2a, at which point the right-side TENG unit is closed, and the transferred charge reaches its maximum value again. With further rotation of the rotary axis, the triboelectric material layers of the TENG units begin to separate, and the transferred charge of the right TENG unit gradually decreases to 0. Eventually, the rotary axis rotates another 180°, returning to state (I) in Figure 2a, at which point the TENG unit partially closes again, and the transfer charge quantity reaches its maximum value, completing the cycle. Through this periodic opening, closing, and separation, the TENG unit facilitates the continuous transfer of charges, effectively promoting the generation of electric current.

The magnet and coil form the EMG unit. Figure 2e illustrates the magnetic field distribution of the EMG based on Faraday’s law of electromagnetic induction, with permanent magnets arranged in an alternating N-S pattern. When the magnet approaches the coil, an induced current is generated in the coil. As the magnet rotates to align with the coil, the induced current in the coil disappears. When the magnet moves away from the coil, the coil generates a reverse induced current. This process repeats continuously, and the coil generates an alternating induced current.

### 3.2. Optimization of TENG Unit Performance for RSM-HNG

**Figure 3:**
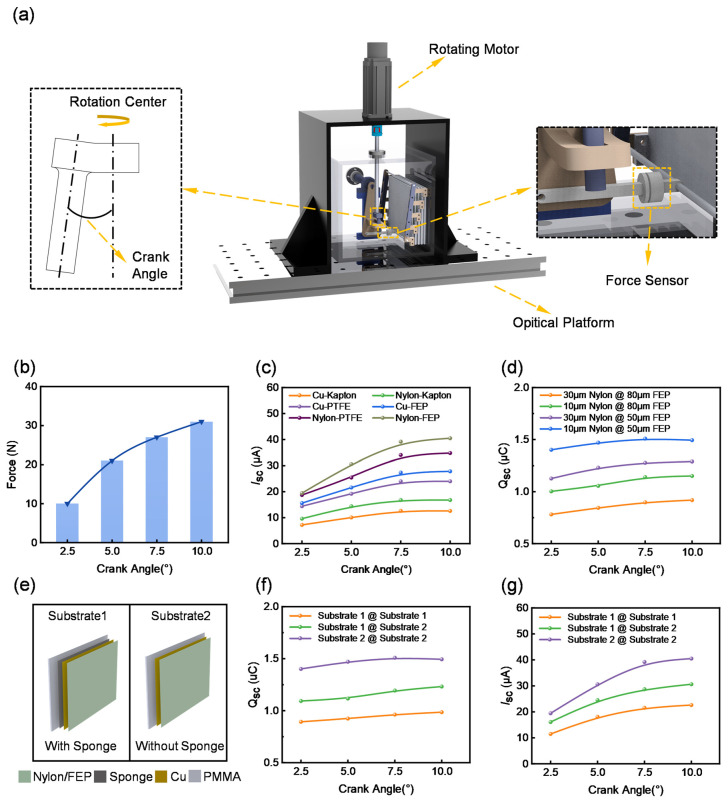
Performance optimization of the RSM-HNG device: (**a**) schematic diagram of the dynamic push–pull force measurement system used for performance optimization of the RSM-HNG; (**b**) relationship between crank angle and push-pull force; (**c**) effect of crank angle on short-circuit current (ISC) for different combinations of triboelectric material types; (**d**) effect of crank angle on QSC for different combinations of triboelectric material thicknesses; (**e**) comparison of two different substrate structures; (**f**) effect of crank angle on QSC for different combinations of substrate structures; (**g**) effect of crank angle on ISC for different combinations of substrate structures.

To optimize the electrical output performance of the TENG unit in the RSM-HNG, a tension testing system was established to measure the tension force at various crank angles in the motion conversion mechanism and its impact on the output performance. As shown in Figure 3a, the test rig consists of a rotary motor, an optical stage, the TENG unit of the RSM-HNG, a tension transducer, and a motor mount. By using a rotary motor in place of a wind cup, we can test the TENG output under constant speed and constant torque conditions, thereby optimizing the performance of the TENG unit. Since the crank angle influences the unfolding angle of the TENG unit, and different unfolding angles result in varying effects on the TENG output, four cranks with angles of 2.5°, 5°, 7.5°, and 10° were fabricated for comparative experiments.

The relationship between the crank angle and the pull force required to drive the TENG unit was first investigated. As shown in Figure 3b, when the crank angle increased, the deployment angle of the TENG unit also increased, thereby requiring a greater driving force. Subsequently, we measured the short-circuit current of the TENG unit at different crank angles with various combinations of triboelectric material. Cu and nylon were selected as the positive triboelectric materials, with copper serving not only as an electrode but also as a triboelectric material layer. polyimide (Kapton), polytetrafluoroethylene (PTFE), and fluorinated ethylene propylene (FEP) were chosen as the negative triboelectric materials. The test results are shown in Figure 3c. As the crank angle increases, the short-circuit currents of the TENG units with different combinations of triboelectric materials exhibit an increasing trend. However, the currents tend to saturate within the range of 7.5° to 10°. Considering the significant increase in the required pulling force as the crank angle increased from 7.5° to 10°, we selected 7.5° as the optimal crank angle. Further analysis revealed that the combination of Nylon and FEP provided the best output at different crank angles. Figure 3d demonstrates the transferred charges for different combinations of triboelectric material layer thicknesses, and we chose 10 μm-thick nylon and 50 μm-thick FEP as triboelectric materials. Since the output performance of the contact–separation TENG is closely related to the degree of contact-separation, PMMA, being a hard material, may result in a rigid contact between the triboelectric material layers. Such a rigid contact could lead to incomplete contact-separation of the triboelectric material layers, thereby affecting the output efficiency of the TENG unit. Therefore, we investigated the output of the TENG unit in both cases, with and without the sponge. As shown in Figure 3e, two substrates were designed: substrate 1 did not incorporate a sponge, while substrate 2 had a sponge placed beneath the electrode. Figure 3f,g displays the transferred charges and short-circuit current measurements under different crank angles. The types of substrate combinations tested included: substrate 1 with substrate 1, substrate 1 with substrate 2, and substrate 2 with substrate 2. The experimental results show that the combination of substrate 1 and substrate 1 exhibits the best output performance, regardless of the crank angle. To further optimize the output performance of the RSM-HNG, a 1 mm-thick sponge was applied as a buffer layer beneath the electrodes of both friction layers. This effectively enhances the contact-separation of the TENG unit, thereby improving the overall output performance of the RSM-HNG.

### 3.3. Electrical Output Performance Analysis of RSM-HNG

**Figure 4:**
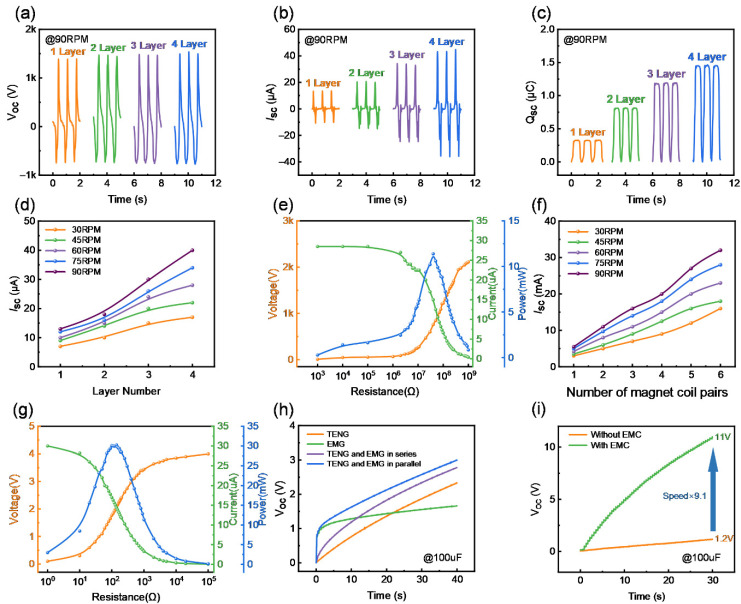
Electrical output performance of the RSM-HNG: (**a**) open-circuit voltage (VOC) curves; (**b**) short-circuit current (ISC) curves; (**c**) transferred charge (QSC) curves of the TENG unit with different numbers of stacked layers at 90 RPM; (**d**) short-circuit current (ISC) of the TENG unit with different numbers of layers at various RPM inputs; (**e**) peak voltage, peak current, and peak power; (**f**) short-circuit current (ISC) of the EMG unit with different numbers of layers under various rotational speed input conditions; (**g**) peak voltage, peak current, and peak power of the EMG unit with a magnet and coil pair number of 6 under different load resistances at 90 RPM; (**h**) charging curves of the RSM-HNG for a 100 µF capacitor; (**i**) comparison of the charging rate of a 100 µF capacitor by the TENG unit with and without energy management circuitry connected.

After the structural design and performance optimization of the RSM-HNG, its overall output performance was tested. To systematically characterize the output performance of the RSM-HNG, a rotary motor was used instead of the wind cup to evaluate the output performance of both the TENG unit and the EMG unit under the influence of various parameters. Figure S4 in the Supplementary Material shows the schematic diagram of the electrical output performance test bench for the RSM-HNG. As shown in Figure 4a–c, the effects of different stacked layers on the open-circuit voltage, short-circuit current, and transferred charges of the TENG unit driven by the rotary motor at 90 rpm are presented. It can be observed that, at the same rotational speed, the open-circuit voltage remains at approximately 2410 V as the number of stacked layers of the TENG unit increases from 1 to 4 layers, while the short-circuit current and the transferred charge increase. When the number of stacked layers reaches 4, the short-circuit current of the TENG unit reaches 40 µA, and the transferred charge is 1.5 µC. The output performance of the TENG units at different speeds was also investigated. Figure S5 in the Supplementary Material illustrates the open-circuit voltage, short-circuit current, and transferred charge of four stacked TENG units at different speeds. It can be observed that the open-circuit voltage and the transferred charge remain nearly constant, while the short-circuit current increases as the speed is raised from 30 rpm to 90 rpm.

Figure 4d illustrates the short-circuit current at different rotational speeds and for different numbers of stacked layers. It can be observed that, with the increase in rotational speed and the number of stacked layers, the short-circuit current exhibits a significant upward trend. The output power of the TENG unit is dependent on the external load. Figure 4e investigates the relationship between the optimum load resistance and the peak output power at a speed of 90 rpm. As the load resistance increases, the load voltage rises from zero to the maximum output voltage of the TENG unit, while the load current decreases from the maximum output current to zero. According to the calculations, the maximum peak power of the TENG unit is 11.7 mW with an optimum load resistance of 40 MΩ. As shown in Figure 4f, the effect of the number of pairs of EMG magnet coils and rotational speed on the short-circuit current of the EMG is presented. As the number of pairs of magnet coils and rotational speed increase, the short-circuit current of the EMG illustrates an increasing trend. The short-circuit current reaches 40 mA when the number of pairs of magnet coils is 6 and the rotational speed is 90 rpm. Figure S6 in the Supplementary Material illustrates the typical waveforms of open-circuit voltage and short-circuit current for six pairs of magnet coils at different rotational speeds. Both the open-circuit voltage and short-circuit current of the EMG show an increasing trend with rising rotational speed, with the open-circuit voltage reaching 4 V at 90 rpm. The optimal load resistance and peak output power of the EMG at 90 rpm are also investigated, as shown in Figure 4g. The load voltage of the EMG increases with load resistance, while the short-circuit current decreases. According to the calculations, the load power reaches its maximum at 140 Ω, with a peak output power of 30.24 mW.

To better investigate the electrical output performance of the RSM-HNG, the charging capacity of the RSM-HNG and the optimal connection between the TENG unit and the EMG unit were studied at 90 rpm. As shown in Figure 4h, the EMG unit rapidly charges a 100 μF capacitor to 1.2 V, but the voltage then increases slowly. The capacitor voltage reaches 1.5 V, while the TENG unit charges the 100 μF capacitor to 2.2 V steadily within 40 s, with the voltage continuing to increase gradually over time. When the TENG unit and EMG unit are connected in parallel and series, the charging voltage of the capacitor reaches 2.8 V and 2.9 V within 40 s, respectively. This indicates that the charging efficiency of the RSM-HNG is slightly higher in parallel connection than in series connection. In parallel connection, the rectified TENG and EMG units each provide a stable voltage and supply current independently to the load, which allows the capacitor to charge more efficiently, with each component operating under its optimal working condition, thereby improving the overall system output efficiency. In contrast, in a series configuration, although the rectified voltages are summed, any decrease in the efficiency of a single component affects the performance of the entire system, as the current must pass through all components. This also demonstrates that the RSM-HNG effectively combines the advantages of both the TENG and EMG units to achieve improved energy-harvesting efficiency, highlighting the potential of the RSM-HNG to power small electronic devices. To enable efficient energy transfer in the RSM-HNG, an electricity management circuit (EMC) [34] is introduced. Figure S7a in the Supplementary Material shows the circuit diagram. This circuit consists of a half-bridge rectifier, a 25 pF high-voltage snubber capacitor, a continuity diode, and an inductor. As shown in Figure 4i, by using EMC, the TENG unit charges a 100 μF capacitor from 1.2 V to 11 V in 30 s at 60 rpm, achieving a 9.1-fold increase in charging capacity efficiency. This demonstrates that the EMC enhances the practical application potential of the device. Simultaneously, we conducted a 12,000-cycle test to verify the durability and output stability of the RSM-HNG. Figure S8 in the Supplementary Material shows the results, which demonstrate good durability and stability.

### 3.4. Application Demonstration of the RSM-HNG

**Figure 5:**
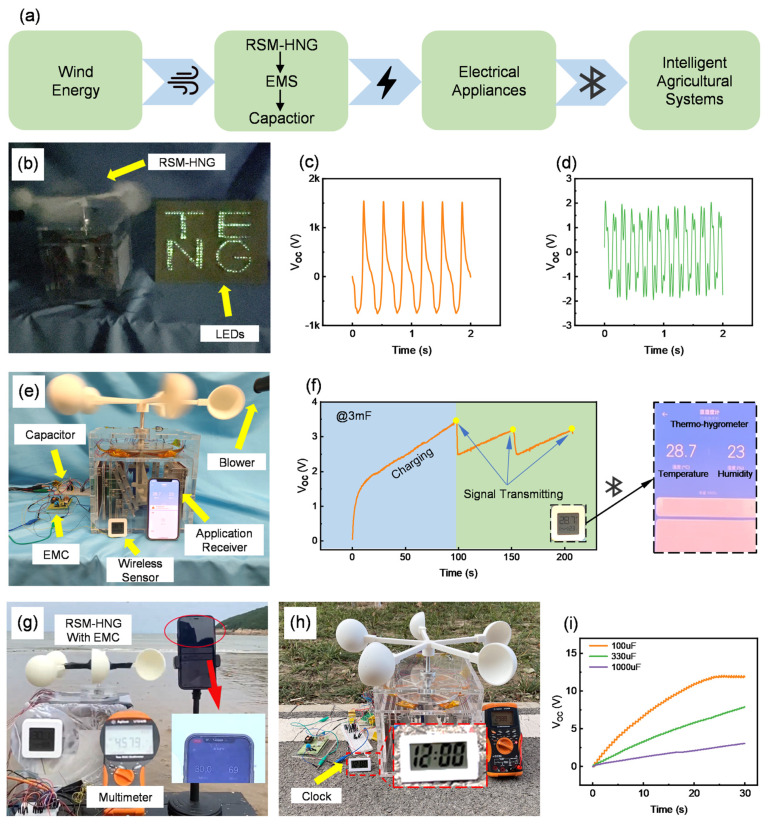
Demonstration of the application of RSM-HNG in smart agriculture: (**a**) schematic diagram of the smart agriculture system; (**b**) photograph of the RSM-HNG powering the LED sign; (**c**) voltage response curve of the TENG unit under a wind speed of 21.5 m/s; (**d**) voltage response curve of the EMG unit under a wind speed of 21.5 m/s; (**e**) photograph of the wireless temperature and humidity sensing system in the smart agriculture setup under the simulated condition of a blower fan in the laboratory; (**f**) capacitance–voltage variation curve of the RSM-HNG when powering a Bluetooth temperature and humidity sensor by charging a 3 mF capacitor; (**g**) photograph of the RSM-HNG driving a Bluetooth thermo-hygrometer in a sea ranch environment; (**h**) photograph of the RSM-HNG driving an electronic clock in a field environment; (**i**) capacitance curves of different capacitor capacitances charged by the RSM-HNG in the EMC setup.

To realize a smart agriculture monitoring system powered by wind energy harvesting, a self-powered and wireless sensing system is developed. Figure 5a shows the system schematic diagram. The RSM-HNG, placed in agricultural and animal husbandry environments, generates cyclic alternating current (AC) driven by wind energy. The power management circuit integrates the AC from both the TENG unit and the EMG unit. The capacitor supplies power to the system, enabling it to drive the intelligent agricultural monitoring system, which performs temperature and humidity monitoring, time display, and road guidance in the agricultural environment.

To verify the practical applicability of the RSM-HNG, a series of tests was conducted using a blower to simulate a wind energy environment. As shown in Figure 5b, driven by wind energy, the RSM-HNG successfully illuminated LED light signs, providing effective road guidance for power-deficient farmland or pastureland (Supplementary Materials, Movie S1). This significantly enhanced the safety of ridge roads, particularly in nighttime conditions. To further validate the potential application of the RSM-HNG in agricultural and livestock environments, a Bluetooth-based ambient temperature and humidity monitoring system was developed. Figure S8b illustrates the schematic of the Power Management Circuit (PMC) utilized in the system. This system was tested to drive the Bluetooth temperature and humidity sensors, with the RSM-HNG powered by a blower. As shown in Figure 5c,d, the voltage response curves of the TENG unit and the EMG unit under a wind speed of 21.5 m/s demonstrate their output frequencies under constant wind speed conditions. Figure 5e illustrates a physical diagram of the RSM-HNG-based Bluetooth temperature and humidity monitoring system, demonstrating its potential application in environmental monitoring. Through actual operation tests, it was found that the RSM-HNG can charge a 3 mF capacitor to 3.3 V under a wind speed of 21.5 m/s, successfully reaching the operating voltage required for the Bluetooth temperature and humidity sensor. As shown in Figure 5f, the charging process lasted approximately 100 s. When the capacitor was charged to 3.3 V, the Bluetooth temperature and humidity sensor began operating normally and transmitted real-time ambient temperature and humidity data to the application receiver via Bluetooth. After the data transfer was complete, the capacitor’s voltage dropped to 2.5 V, at which point the RSM-HNG resumed charging the capacitor. In about 50 s, the capacitor voltage reached a sufficient level to restart the Bluetooth temperature and humidity sensor, and the data was successfully transmitted. This experimental result demonstrates the potential of the RSM-HNG for environmental temperature and humidity monitoring in agriculture and animal husbandry. By effectively utilizing ambient wind energy, the RSM-HNG provides stable power to low-power devices such as Bluetooth temperature and humidity sensors. This highlights the significant potential of the RSM-HNG for remote environmental monitoring in applications such as agriculture and livestock management.

To further validate the RSM-HNG’s applicability in different environments, it was deployed in a sea ranch and a field road environment, utilizing ambient wind energy for operation. In these environments, the RSM-HNG demonstrated strong energy-harvesting capability and stable performance. As shown in Figure 5g, the RSM-HNG is deployed in a sea ranch environment integrated with an ambient temperature and humidity monitoring system. Powered by the sea breeze, the RSM-HNG successfully charged a 3 mF capacitor to 4.5 V after 3 min of operation, reaching the required operating voltage for the Bluetooth temperature and humidity sensor. At this point, the Bluetooth sensor activated and began transmitting real-time ambient temperature and humidity data to the application receiver via Bluetooth. This experimental result demonstrates the efficient energy-harvesting capability of the RSM-HNG in a sea ranch environment, providing sustainable power support for remote environmental monitoring (Supplementary Material, Movie S2). We have also verified the ability of the RSM-HNG to drive an electronic clock when powered by wind energy (Supplementary Material, Movie S3). Figure 5f demonstrates that in a field environment, the RSM-HNG, powered by ambient wind energy, charges a 3 mF capacitor to approximately 3 V in 3 min. This voltage is sufficient to power an electronic clock for time display in a farmland. These results show that even in a power-deficient agricultural environment, the RSM-HNG can provide basic time display services to local residents, significantly enhancing farm management and work efficiency.

To thoroughly evaluate the energy harvesting efficiency of the RSM-HNG, experiments were conducted under a wind speed of 21.5 m/s, using three capacitors with different capacitances (100 μF, 330 μF, and 1000 μF) for the charging test. By monitoring the charging process of these capacitors, the energy-harvesting capability of the RSM-HNG under different load conditions can be visually assessed. As shown in Figure 5i, the experimental results indicate that the RSM-HNG efficiently charges a 100 μF capacitor to 12 V, a 330 μF capacitor to 7.4 V, and a 1000 μF capacitor to 1.5 V in just 30 s. These results demonstrate that the RSM-HNG exhibits strong energy-harvesting capability and can charge capacitors of different capacitances in a relatively short period.

## 4. Conclusions

In this study, we present the hybrid nanogenerator based on a rotational-swinging mechanism (RSM-HNG) as an innovative solution for wind energy harvesting, enabling the establishment of an intelligent agricultural monitoring system. The RSM-HNG effectively combines the triboelectric nanogenerator (TENG) and electromagnetic generator (EMG) to achieve efficient energy harvesting. The system converts rotational motion driven by wind energy into a swinging motion, facilitated by a parallelogram mechanism. This design allows for the simultaneous contact–separation of multiple stacked layers of TENG, enhancing the service life of the system compared to conventional wind energy-harvesting methods.

Upon stacking four layers, the short-circuit current of the TENG increased from 16 μA to 40 μA, with a corresponding increase in the transferred charge. Furthermore, the system successfully powers a self-sustained wireless temperature and humidity monitoring system, an electronic clock driver, and a road guide, all powered by harvested wind energy. The wireless temperature and humidity monitoring system transmits data every 50 s. These findings demonstrate that the RSM-HNG, driven by wind energy, provides a continuous power supply for intelligent agricultural and livestock monitoring systems, as well as environmental monitoring applications. The system is particularly valuable in areas with limited access to electricity but abundant wind energy resources, offering broad potential for applications in agriculture and ranching.

## Data Availability

The data presented in this study are available on request from the corresponding author due to intellectual property.

## Supplementary Materials

The following supporting information can be downloaded at: https://www.mdpi.com/article/10.3390/s25165041/s1, Figure S1: Physical photograph of hybrid nanogenerator based on rotational-swinging mechanism (RSM-HNG). Figure S2: Main dimension drawing of RSM-HNG. (a) Sectional view of the main configuration of the RSM-HNG, highlighting its primary physical dimensions. (b) Length dimensions of the RSM-HNG and the sectioning direction in the main view. Figure S3: Schematic structure of the Electromagnetic Generator (EMG) unit. (a) Exploded view of the structure of the EMG unit. (b) Schematic diagram of the magnet arrangement and the direction of coil winding in the EMG unit. Figure S4: Schematic diagram of the electrical output performance test bench for the RSM-HNG. Figure S5: Electrical output performance of TENG unit at different rotational speeds. (a) Open-circuit voltage (VOC), (b) short-circuit current (ISC), and (c) transferred charges (QSC). Figure S6: Electrical output performance of EMG unit at different rotational speeds. (a) Open-circuit voltage (VOC), (b) short-circuit current (ISC). Figure S7: Schematic diagram of the circuit used by RSM-HNG. (a) Schematic diagram of the energy management circuit of the TENG unit. (b) Circuit schematic diagram of RSM-HNG to drive a load by power management circuit (PMC). Figure S8: Stability testing of the TENG unit of the RSM-HNG. Figure S9: Humidity testing of the TENG unit of the RSM-HNG. Figure S10: Temperature testing of the TENG unit of the RSM-HNG. Figure S11: The wiring method of TENG unit. Table S1. Performance comparison of different energy harvesting devices. Figure S12: The wiring method of TENG unit. Theoretical formula derivation of TENG. Movie S1: RSM-HNG Lights LEDs. Movie S2: RSM-HNG Powers a Bluetooth Temperature and Humidity Sensor. Movie S3: RSM-HNG Powers an Electronic Clock.
