# Supplementary material for: A Hybrid Nanogenerator Based on Rotational-Swinging Mechanism for Energy Harvesting and Environmental Monitoring in Intelligent Agriculture
Source: Sensors (Basel). 2025 Aug 14;25(16):5041. doi: 10.3390/s25165041 (PMC12389822; doi:10.3390/s25165041)
Supplement: Supplementary file 1 [file sensors-25-05041-s001.zip › Supplementary Material.pdf]

Supplementary Material

A Hybrid Nanogenerator Based on Rotational-Swinging Mechanism for Energy Harvesting and Environmental Monitoring in Intelligent Agriculture

The PDF file includes:

Fig. S1: Physical photograph of hybrid nanogenerator based on rotational-swinging mechanism (RSM-HNG).

Fig. S2: Main dimension drawing of RSM-HNG. (a) Sectional view of the main configuration of the RSM-HNG, highlighting its primary physical dimensions. (b) Length dimensions of the RSM-HNG and the sectioning direction in the main view.

Fig. S3: Schematic structure of the Electromagnetic Generator (EMG) unit. (a) Exploded view of the structure of the EMG unit. (b) Schematic diagram of the magnet arrangement and the direction of coil winding in the EMG unit.

Fig. S4: Schematic diagram of the electrical output performance test bench for the RSM-HNG.

Fig. S5: Electrical output performance of TENG unit at different rotational speeds. (a) Open-circuit voltage (V_{OC}), (b) short-circuit current (I_{SC}), and (c) transferred charges (Q_{SC}).

Fig. S6: Electrical output performance of EMG unit at different rotational speeds. (a) Open-circuit voltage (V_{OC}), (b) short-circuit current (I_{SC}).

Fig. S7: Schematic diagram of the circuit used by RSM-HNG. (a) Schematic diagram of the energy management circuit of the TENG unit. (b) Circuit schematic diagram of RSM-HNG to drive a load by power management circuit (PMC).

Fig. S8: Stability testing of the TENG unit of the RSM-HNG.

Fig. S9: Humidity testing of the TENG unit of the RSM-HNG.

Fig. S10: Temperature testing of the TENG unit of the RSM-HNG.

Fig. S11: The wiring method of TENG unit.

TableS1. Performance comparison of different energy-harvesting devices.

Fig. S12: The wiring method of TENG unit.

Theoretical formula derivation of TENG

Other Supplementary Material for this manuscript includes the following:

Movie S1: RSM-HNG Lights LEDs. (MP4)

Movie S2: RSM-HNG Powers a Bluetooth Temperature and Humidity Sensor. (MP4)

Movie S3: RSM-HNG Powers an Electronic Clock. (MP4)

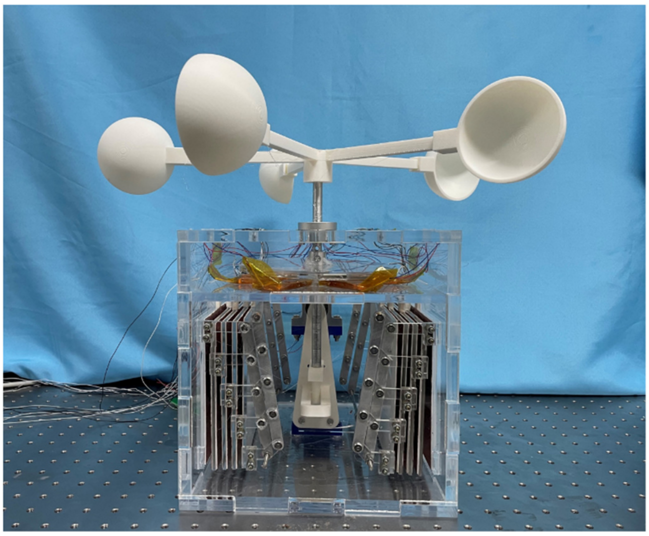

Fig. S1: Physical photograph of hybrid nanogenerator based on rotational-swinging mechanism (RSM-HNG).

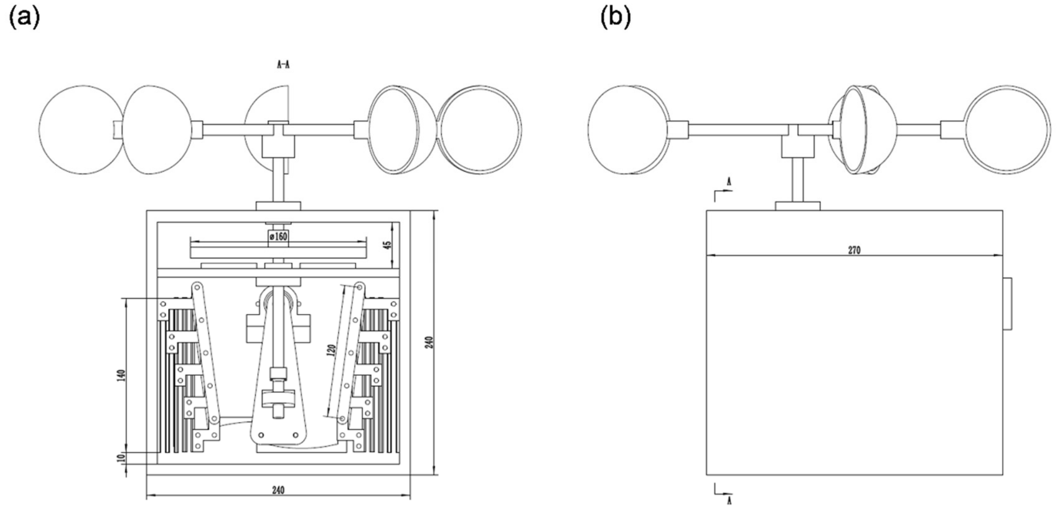

Fig. S2: Main dimension drawing of RSM-HNG. (a) Sectional view of the main configuration of the RSM-HNG, highlighting its primary physical dimensions. (b) Length dimensions of the RSM-HNG and the sectioning direction in the main view.

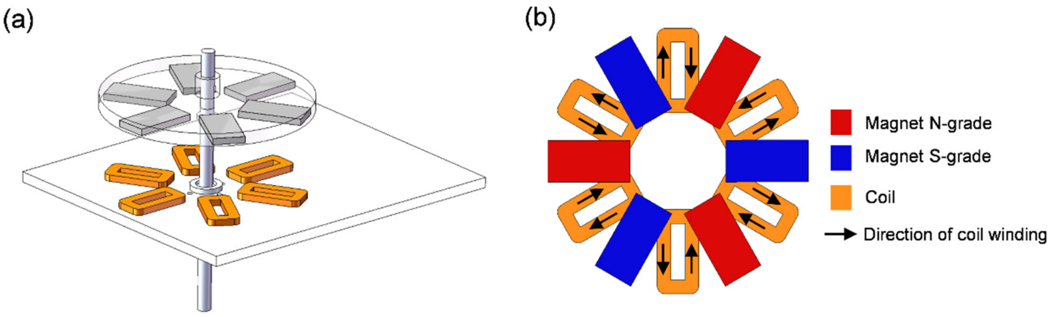

Fig. S3: Schematic structure of the Electromagnetic Generator (EMG) unit. (a) Exploded view of the structure of the EMG unit. (b) Schematic diagram of the magnet arrangement and the direction of coil winding in the EMG unit.

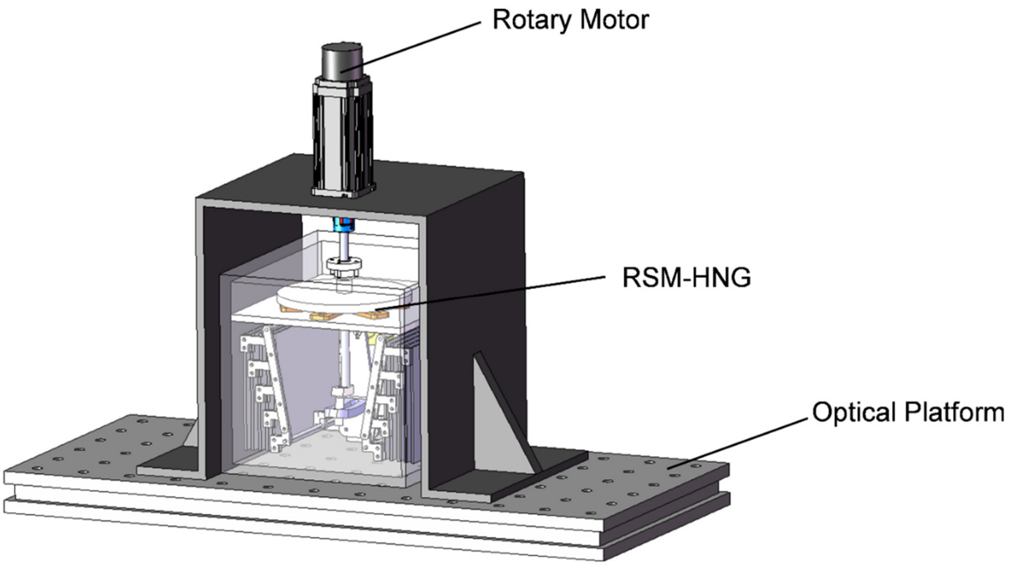

Fig. S4: Schematic diagram of the electrical output performance test bench for the RSM-HNG.

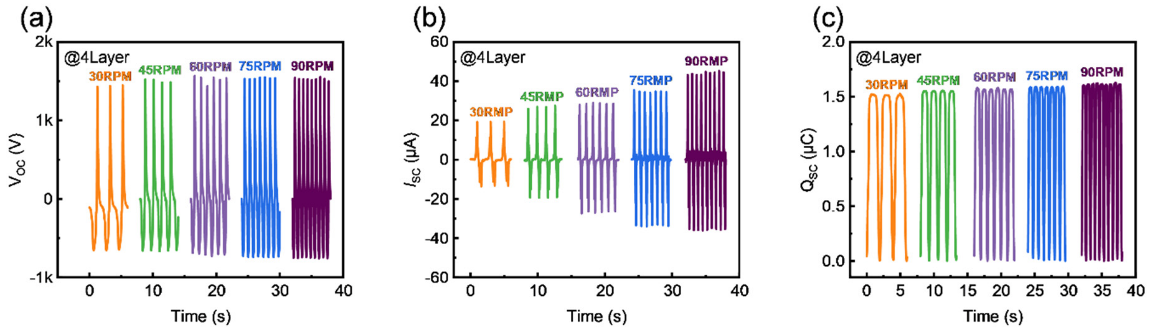

Fig. S5: Electrical output performance of TENG unit at different rotational speeds. (a) Open-circuit voltage (V_{OC}), (b) short-circuit current (I_{SC}), and (c) transferred charges (Q_{SC}).

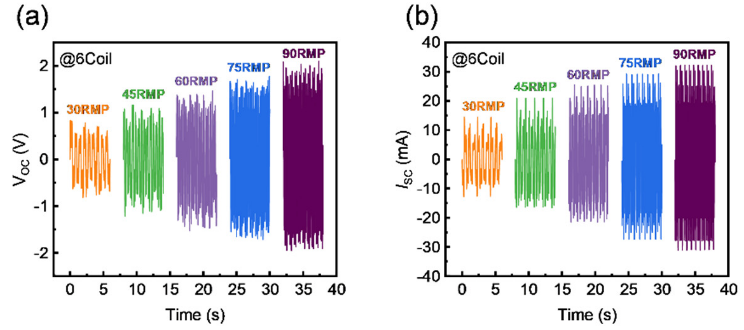

Fig. S6: Electrical output performance of EMG unit at different rotational speeds. (a) Open-circuit voltage (V_{OC}), (b) short-circuit current (I_{SC}).

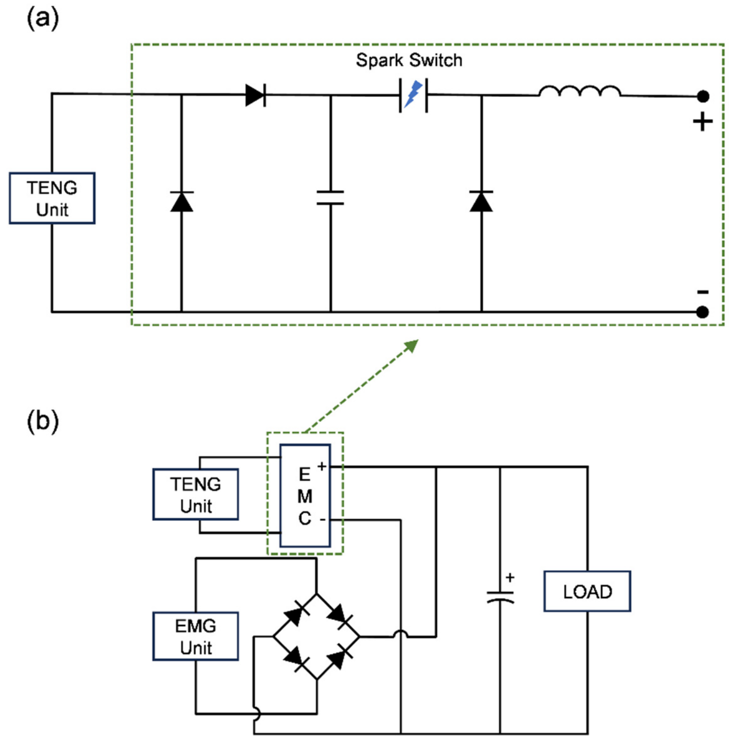

Fig. S7: Schematic diagram of the circuit used by RSM-HNG. (a) Schematic diagram of the energy management circuit of the TENG unit. (b) Circuit schematic diagram of RSM-HNG to drive a load by power management circuit (PMC).

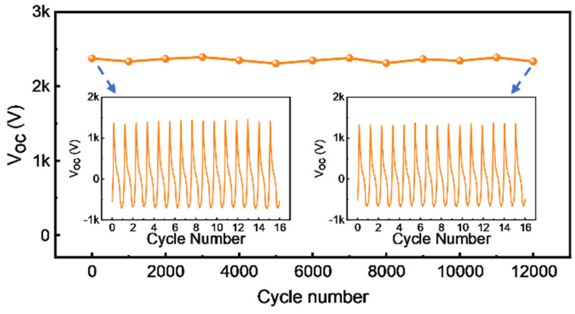

Fig. S8: Stability testing of the TENG unit of the RSM-HNG.

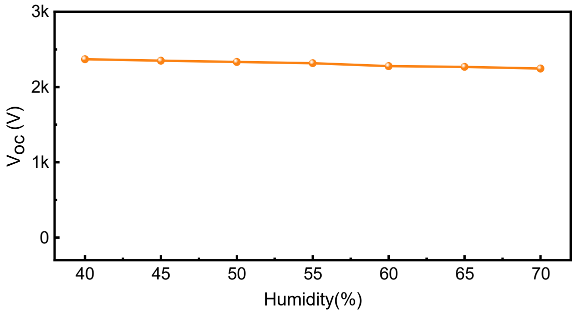

Fig. S9: Humidity testing of the TENG unit of the RSM-HNG.

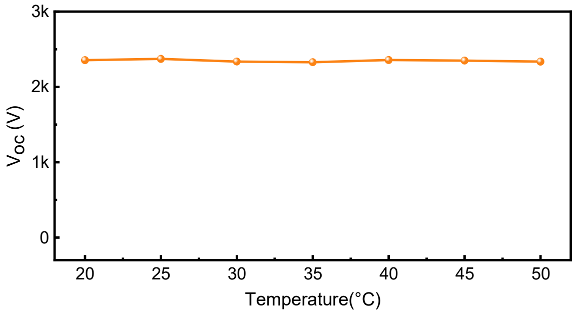

Fig. S10: Temperature testing of the TENG unit of the RSM-HNG.

Reported work	TENG 功率
MLS-TENG[1]	10.57mW
TEE-HEH[2]	3mW
TEHG[3]	0.3mW
RSM-HNG(Our Work)	11.7mW

TableS1. Performance comparison of different energy-harvesting devices

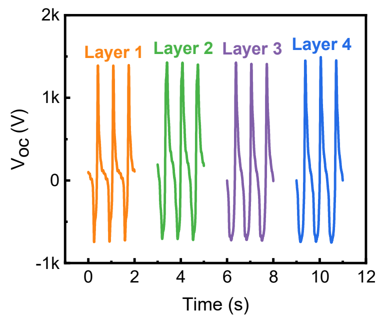

Fig. S11: Open-circuit voltage of each layer in TENG unit.

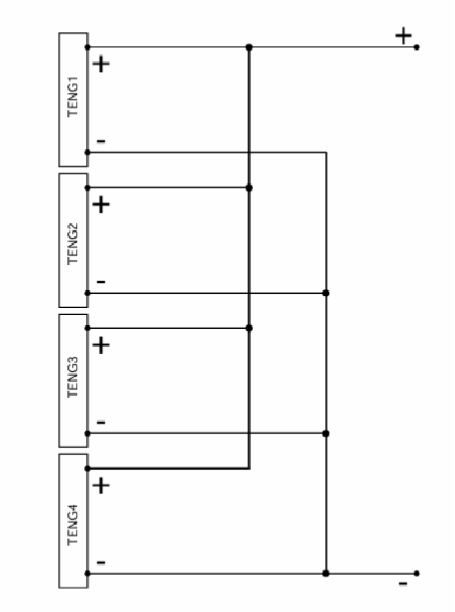

Fig. S12: The wiring method of TENG unit.

Theoretical formula derivation of TENG:

The contact–separation type triboelectric nanogenerator utilizes the triboelectric effect and electrostatic induction principles. Through periodic contact–separation, the triboelectric nanogenerator continuously converts mechanical energy into electrical energy. Based on the assumption of an infinitely large plate, the charges on the material surface are uniformly distributed. The potential difference can be derived by considering the charge distribution during separation, as described by Gauss's law, which is as follows:

$$\Phi_E = \oint \vec{E} \cdot d\vec{A} = \frac{Q_{\text{enc}}}{\varepsilon_0} \tag{S1}$$

Let the charge on the material surface be Q and the area of the triboelectric layer material be A. The electric field strength can be derived from Gauss's law as follows:

$$E = \frac{Q}{\varepsilon_0 A} \tag{S2}$$

The open-circuit voltage equation can be derived as follows:

$$V_{OC} = E \cdot x(t) = \frac{Q}{\varepsilon_0 A} x(t) \tag{S3}$$

In the equation, Q is the charge on the material surface, ε0 is the vacuum permittivity, A is the area of the dielectric plane, and x(t) is the distance between the dielectrics.

Since the distance between the two electrodes of the contact–separation type triboelectric nanogenerator is a function of time x(t), and considering the effective thickness d0 of the dielectric layer, the equivalent capacitance of the contact–separation type triboelectric nanogenerator can be expressed as:

$$C(x) = \frac{\varepsilon_0 A}{d_0 + x(t)} \tag{S4}$$

For the transferred charge, it can be calculated using the charge formula for parallel plate capacitors. The charge formula is:

$$Q_{SC} = C(x) \times V_{OC} \tag{S5}$$

By substituting equations (S3) and (S5), the formula for the transferred charge can be obtained:

$$Q_{SC} = \frac{Qx(t)}{d_0 + x(t)} \tag{S6}$$

According to equation (S6), it can be concluded that the transferred charge of the contact-separation type triboelectric nanogenerator is proportional to the charge on the material surface. During the contact–separation process, when the surfaces of two different materials come into contact, charge transfer occurs between the material surfaces. The amount of charge transferred mainly depends on the accumulation of surface charge. The larger the surface charge, the greater the amount of charge that can be transferred during contact. As the number of layers in the triboelectric nanogenerator unit increases, the overall surface charge of the generator unit also increases.

Next, we derive the short-circuit current. According to the definition of current, current is the rate of change of charge. The formula for the short-circuit current is:

$$I_{SC} = \frac{dQ_{SC}}{dt} = \frac{Qv(t)d_0}{\left[d_0 + x(t)\right]^2} \quad \text{(S7)}$$

In the equation, A is the area of the dielectric plane, Q is the surface charge of the material, v(t) is the contact-separation speed, d0 is the effective thickness of the dielectric layer, and x(t) is the distance between the dielectrics.

From equation (S7), it can be seen that the short-circuit current of the triboelectric nanogenerator is closely related to multiple factors. The surface charge on the material plays a decisive role in the magnitude of the current. The larger the surface charge, the more charge is generated, resulting in a larger short-circuit current. As the number of layers in the triboelectric nanogenerator unit increases, the overall surface charge of the generator unit increases, and the short-circuit current also increases.

References

1 H. Zhou, X. Wei, B. Wang, E. Zhang, Z. Wu and Z. L. Wang, Adv Funct Materials, 2023, 33, 2210920.

2 Q. Wang, D. Hu, X. Huang, Z. Chen, Z. Yuan, L. Zhong, Q. Sun, F. Wang, S. Xu and S. Chen, Advanced Energy Materials, 2024, 2403931.

3 J. Zhao, J. Mu, H. Cui, W. He, L. Zhang, J. He, X. Gao, Z. Li, X. Hou and X. Chou, Adv Materials Technologies, 2021, 6, 2001022.
